# Microcrystalline Cellulose Extracted from Native Plants as an Excipient for Solid Dosage Formulations in Drug Delivery

**DOI:** 10.3390/nano10050975

**Published:** 2020-05-19

**Authors:** Camila Viera-Herrera, Javier Santamaría-Aguirre, Karla Vizuete, Alexis Debut, Daniel C. Whitehead, Frank Alexis

**Affiliations:** 1 School of Biological Sciences and Engineering, Yachay Tech University, Urcuquí 100650, Ecuador; camila.viera@yachaytech.edu.ec; 2 Faculty of Chemical Sciences, Universidad Central del Ecuador, Instituto de Investigación en Salud Pública y Zoonosis (CIZ), Quito 170130, Ecuador; jrsantamaria@uce.edu.ec; 3 Center of Nanoscience and Nanotechnology, Universidad de las Fuerzas Armadas ESPE, Sangolquí 170501, Ecuador; ksvizuete@espe.edu.ec (K.V.); apdebut@espe.edu.ec (A.D.); 4 Department of Chemistry, Clemson University, Clemson, SC 29634, USA; dwhiteh@clemson.edu

**Keywords:** pharmaceutical excipient, cellulose, tablets, drug delivery

## Abstract

Excipients represent the complement of the active principle in any pharmaceutical form. Their function is to provide stability, protection, and to ensure absorption of the drug and acceptability in patients. Cellulose is a conventional excipient in many pharmaceutical solid dosage products. Most of the sources used to extract microcrystalline cellulose come from cotton or wood, which are expensive and in high demand from other industries. As plants are considered the main source of excipient production, we have taken advantage of the biodiversity of Ecuador to evaluate microcrystalline cellulose extracted from borojó (*Alibertia patinoi*), a native plant, as an excipient for solid dosage formulations. The method of choice for tablet manufacturing was direct compression since it is a conventional fabrication method in the pharmaceutical industry. First, we performed scanning electron microscopy (SEM), Fourier-transform infrared (FTIR) spectroscopy, and X-ray diffraction (XRD) in order to compare the structure and characteristics of the extracted cellulose with two reference commercial cellulose materials. Second, we performed quality tests to evaluate the use of the isolate as an excipient including fluidity, hardness, friability, and disintegration. Compared with commercial and microcrystalline cellulose, the extracted cellulose from the native plant showed comparable characteristics and is consequently a potential excipient that could be used in the pharmaceutical industry. Last, we performed a dissolution test in which we concluded that all tablets have a short release time of active principle.

## 1. Introduction

Among all pharmaceutical dosage products, oral solids are the most commonly used by consumers to treat diseases or relieve pain. These forms are obtained by different methods that involve mixing the active ingredient with adequate excipients to obtain a solid product such as a capsule, tablet, or chewable tablet [1]. Excipients are the non-active part of the formulation that support the drug delivery system during its manufacture by protecting the active ingredient and enhancing stability. When the pharmaceutical form is administered, its function is to improve patient acceptability by masking a displeasing taste or texture and ultimately to assure physiological absorption of the drug [2,3,4].

The main source of excipients are plants, from which polymers can be extracted [5]. Vegetable oils, fatty acids, and carbohydrates (for example, sucrose, starch, pectin, and cellulose) have been widely investigated [6]. Some of the main reasons to use plant-based components in the pharmaceutical industry are that the raw material is easy to access, it is renewable, and the plants contain those organic substances in sufficient amounts to be used [6]. As a result of this, the process to extract a natural excipient, or even an active principle, is often more cost-effective than producing a synthetic substitute [5]. Moreover, some synthetic compounds have been shown to be toxic compared to plant-based products, which are mainly biodegradable, biocompatible, and non-toxic [5,6,7].

Microcrystalline cellulose (MCC) is the most common cellulose-derived excipient used in the pharmaceutical industry [8]. Cellulose and cellulose-based products are widely used as excipients because their porosity enhances liquid uptake, thus making swelling of the drug product faster and easier [9]. Cellulose works as an immediate, sustained, or delayed release excipient [10].

Cellulose is extracted from the cell walls of many organisms that range from bacteria (cyanobacteria) and prokaryotes (Acetobacter, Rhizobium, Agrobacterium) to eukaryotes (fungi, amoebae, green algae, freshwater and marine algae, mosses, ferns, angiosperms, gymnosperms), and even some animals (tunicates) [11,12,13]. Nonetheless, MCC is usually obtained only from the hydrolysis of cellulose of cotton or wood. As these materials are widely used in furniture, paper, and building and textiles industries, it can be problematic to require them for pharmaceuticals. Consequently, it is necessary to evaluate the performance and cost of other sources for MCC extraction such as herbaceous plants, grass, aquatic plants, fruits, or any material that contains significant amounts of cellulose [14].

New sources of MCC have shown differences in terms of chemical composition and physicochemical properties (crystallinity, moisture content, surface area, porosity, molecular weight, etc.) [15]. This leads to a larger diversity of MCC that can be used in different pharmaceutical applications. The present study aimed to evaluate MCC extracted from a native Ecuadorian plant, borojó, as an excipient for solid dosage formulation. The potential use of cellulose from plants as excipients could have a significant impact on the future of solid dosage in pharmaceutics.

## 2. Materials and Methods 

### 2.1. Cellulose Samples

All experiments described below were performed using three samples: commercial powdered cellulose (named CC; Elcema^®^ G250 brand from Degussa), commercial microcrystalline cellulose (CMCC; Avicel^®^ pH 102), and cellulose isolated from borojó (BC).

### 2.2. BC Extraction

Cellulose from the native Ecuadorian plant borojó (*Alibertia patinoi*) was extracted following established protocols which consist of grinding the samples with an electrical grinder and then extraction with chemicals followed by acid/base treatment, bleaching, and multiple water washings to eliminate residual chemicals [16].

### 2.3. Preparation of BC as an Excipient

CC and CMCC samples were already powdered and ready to be used. In contrast, BC was isolated as a dough. As the excipients should be powdered, BC was dried at 60 °C for 4 h until it became hard. After drying, it was milled with a mortar and pestle to reduce particle size until it was able to be sieved through 850 and 250 μm meshes.

### 2.4. Characterization of Excipients

#### 2.4.1. Scanning Electron Microscopy (SEM)

Samples were frozen using liquid nitrogen and then lyophilized using an ILSHIN BIOBASE freeze dryer. The dried samples were fixed onto aluminum pin holders using double-sided conductive carbon tape and sputter-coated for 60 s with a layer of 20 nm of gold (99.99% purity, Quorum Q150R ES, Quorum Technologies, East Sussex, UK). This technique was carried out using a MIRA 3 field emission scanning electron microscope (FEG-SEM, Tescan Mira 3, Brno, Czech Republic). High resolution and low-noise images of the excipients were obtained on a scale of 200 μm.

#### 2.4.2. X-ray Diffraction (XRD)

XRD information was obtained from an EMPYREAN diffractometer (PANalytical, Almelo, The Netherlands) in a Bragg-Brentano configuration at 40 kV and 45 mA and monochromatic X rays of Cu K-α wavelength (λ = 1.541 Å) using a Ni filter. XRD patterns were obtained over a 2θ angle range of 5°–90°. Once the data was plotted, the crystallinity index (*CrI*) was calculated using Equation (1).

(1) $$CrI\left(\%\right)=\frac{I_{\left(002\right)}-I_{\left(am\right)}}{I_{\left(002\right)}}\times 100\%$$

$I_{\left(002\right)}$. represents the diffraction intensity at 2θ equal or close to 22° representing crystalline material, and $I_{\left(am\right)}$ depicts the diffraction intensity at 2θ equal to 18° representing amorphous material [17].

#### 2.4.3. Fourier-Transform Infrared (FTIR) Spectroscopy

IR spectra were obtained using a Spectrum Spotlight 200 FT-IR instrument (Perkin Elmer, Norwalk, CT, USA). The spectrum of a gold-plated sample holder was collected as a blank. The wavelength was set between 4000 and 500 cm^−1^ with a total of 36 scans and a 4 cm^−1^ wavelength resolution.

### 2.5. Rheological Properties of Excipients

#### 2.5.1. Loss on Drying

The volatile material of the samples was determined using a moisture balance Hx204 (Mettler Toledo, Switzerland).

#### 2.5.2. Carr’s Index and Hausner’s Ratio

For these two tests, a poured volume of each excipient was determined and compared with tapped volume. The poured volume was defined using a graduated cylinder in which the excipient was placed, and the volume marked was written down. The tapped volume was measured after giving the graduated cylinder smooth bumps against a flat surface up to constant volume. Additionally, the mass was weighed with the instrument tared. With these data, the poured density and tapped density was calculated using the following formula.

(2) $$\partial_{poured}=\frac{mass}{poured\;volume}$$

(3) $$\partial_{tapped}=\frac{mass}{tapped\;volume\;}$$

Carr’s index and Hausner’s ratio were obtained applying the following formulae.

(4) $$Carr^{\prime }s\;index\;\left(\%\right)=\frac{\partial_{tapped}-\partial_{poured}}{\partial_{tapped}}\times 100$$

(5) $$Hausner^{\prime }s\;ratio=\frac{\partial_{tapped}}{\partial_{poured}}\times 100$$

The values obtained were evaluated by comparing values with established tables that represent the acceptable values for excellent, good, and poor fluidity [18].

#### 2.5.3. Angle of Repose

A simple test was performed by placing a funnel at approximately 5 cm over a grid sheet. The funnel outlet was covered, the excipient was poured into the funnel, and as a final step, the powder was allowed to flow out of the funnel outlet to form a conical structure [19]. The diameter of the base and the height of the cone were measured, and the angle of repose was calculated using the following formula.

(6) $$\propto =tan^{-1}\left(\frac{2h}{b}\right)$$

The values of the angle of repose for excellent, good, passable, and poor flowability are also established [18].

#### 2.5.4. Statistical Analysis

*R* Studio, a statistical software package, was used to analyze and compare all of the rheological properties in the three samples (i.e., CC, CMCC, and BC). We performed one-way ANOVA, a statistical test that compares the means of 2 or more groups, for each characteristic, in which the null hypothesis was BC = CC = CMCC. As alternative hypothesis we established that the property that was being evaluated for the different cellulose types was not all equal. The model proposed was linear in which the Carr’s index, Hausner’s ratio, and angle of repose were dependent on the type of cellulose that was used.

Once ANOVA results were obtained, the *p*-value was compared with the significance level, which was chosen as 1% or 0.01. A smaller number led to the rejection of the null hypothesis. Tukey multiple comparisons of means with a 95% family-wise confidence level was used to determine which ones were equal. The combination of two (CC-BC, CMCC-BC, or CMCC-CC) that showed a *p*-value greater than significance level meant that they were not statistically significantly different.

### 2.6. Compression

The main types of excipients such as disintegrant, diluent, glidant, and lubricant were included, as seen in Table 1. Acetaminophen was chosen as the active principle as it is one of the most commonly used therapeutics worldwide. A total of 20 g of formulation was made.

Acetaminophen, cellulose and lactose were sifted through the 850 μm mesh and then mixed for 3 min. After that, aerosil was sifted through the 0.25 μm mesh. This was incorporated in the previous blend and mixed again for 2 min. Finally, the same process was followed with PEG 6000.

A Classic Piccola (Riva, Argentine) rotating tableting machine was used for compression, with a force/tension of 4 kN and tablet dimensions of 10.3 mm diameter.

### 2.7. Quality Control of Tablets

#### 2.7.1. Hardness

A Tablet hardness tester KEY model HT-300 was used to measure the resistance of fracture of the tablets. It consisted of a machine made up of two plates that approach each other applying a compressive force. The tablet was placed between them, and when it broke, the force was automatically registered. These data were tabulated to then be analyzed.

#### 2.7.2. Friability

This characteristic was determined using an ERWEKA friabilator. First, six tablets, free of powder, were weighed, placed onto the drum and the time was set for 4 min. After that, the tablets were weighed again. These data were tabulated and used to calculate the percentage of friability, given by Equation (7).

(7) $$\%\;Friability=\frac{W_{i}-W_{f}}{W_{i}}\times 100$$

*W_i_* and *W_f_* represent the initial and final weight, respectively. The result for this calculation should not give more than 1%, otherwise it represents lack of resistance to abrasion and fracture [20].

#### 2.7.3. Disintegration Time

The apparatus, Pharma Test, is composed of two discs holding six glass cylinders covered by a stainless-steel mesh at the bottom. [21]. This, altogether, was added to a container with the corresponding fluid (in this case water) at 37 °C [21].

To initiate testing, a tablet was placed in each hole and the machine was actioned. It started moving up and down with the tablets immersed in the liquid media. The time at which any residue of the tablet remained on the screen was registered in a table to be compared.

#### 2.7.4. Dissolution Test

Based on United States Pharmacopeia (USP), the paddle method was applied. This equipment consisted of six vessels with a capacity of 1000 mL each with a paddle used as the stirring element [22]. Before starting the equipment, water at 37 °C was added to the big container to maintain this temperature in the smaller vessels, simulating body temperature. The dissolution media was 900 mL of 0.05 M monobasic phosphate buffer pH 5.8 at 37 °C. As a preliminary test, two tablets of each type (CC, BC, CMCC) were added, and then the machine was actioned to 50 rpm for 60 min [23].

Over the course of the 60 min sampling period, small amounts of solution were taken out at 5, 10, 20, 30, 45, and 60 min, replacing the volume taken with fresh media [22].

The concentrations of the active principle at each time were determined using ultraviolet-visible (UV-VIS) spectrophotometry using a calibration curve. Then, this was transformed to percentage dissolved. The data obtained was tabulated to then plot the dissolution profile of the tablets made with CC, CMCC, and BC samples.

## 3. Results

### 3.1. Characterization of Excipients

#### 3.1.1. Crystallinity of the Cellulose Excipients

**Figure 1:**
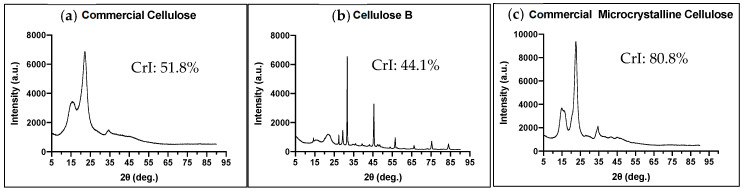
X-ray diffraction and crystallinity index (CrI) for powders of the three excipients. (**a**) commercial cellulose (CC); (**b**) borojó cellulose (BC); (**c**) commercial microcrystalline cellulose (CMCC).

The X-ray diffraction patterns for commercial cellulose (CC), commercial microcrystalline cellulose (CMCC), and borojó cellulose (BC) are shown in Figure 1. It can be seen that in commercial cellulose (CC) there is one prominent sharp peak between 16° and 22°, corresponding to the (101) and (002) lattice diffraction, respectively. The broad peak at 2θ around 35° corresponds to the crystalline behavior of typical cellulose I with ordered regions [24]. In CMCC, the higher peak is also between 20° and 25°, but it is narrower than the previous one, which indicates a higher crystallinity index. The CMCC is also a typical cellulose I. Unlike the previous powders, BC has more sharpened peaks, the bigger is around 28°, followed by one at 46° and others are smaller at 27, 30, 57, 75, and 84°, probably indicating a larger contribution of smaller crystallite sizes. The values of the crystallinity index calculated according to Equation (1) were 44.1% for BC, 51.8% for CC, and 80.8% for CMCC.

#### 3.1.2. Chemical Composition of the Cellulose Excipients

**Figure 2:**
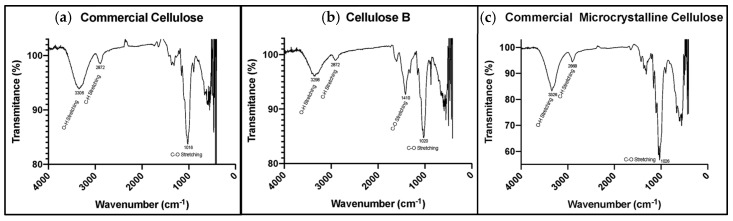
Fourier-transform infrared spectra of powders of the three excipients. (**a**) CC; (**b**) CMCC; (**c**) BC.

The IR spectra for CC, CMCC, and BC can be seen in Figure 2a–c, respectively. A broad peak is shown around 3200–3300 cm^−1^ attributable to O–H stretching. At approximately 2800 cm^−1^ there is a small peak indicating C–H stretching. A narrower peak can be distinguished around 1020 cm^−1^, showing C–O stretching. An additional peak is evident at 1410 cm^−1^, exhibiting C–H bending. All of the peaks represent characteristic groups present in cellulose. Additionally, the IR spectra confirms the absence of impurities or extra components such as hemicellulose or lignin residues in all of the samples.

#### 3.1.3. Morphology of the Cellulose Excipients 

**Figure 3:**
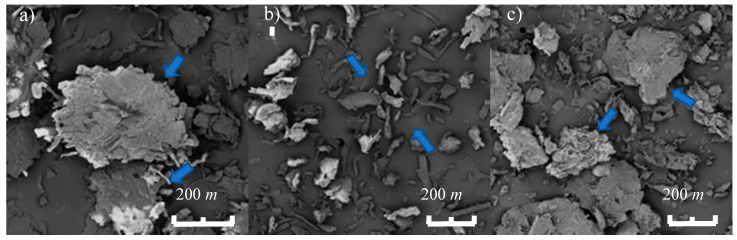
Scanning electron microscopy (SEM) micrographs at scale 200 μm for powders of the different excipients. (**a**) CC; (**b**) CMCC; (**c**) BC.

Figure 3 shows SEM images at 200 μm scale for CC, CMCC, and BC (Figure 3a,c, respectively). Although the particle sizes were not uniform, the microcrystalline cellulose had an approximate diameter of 100 μm. Borojó cellulose had an approximate diameter of 200 μm, and the commercial cellulose sample had a diameter of 400 μm. All three cellulose powders exhibited similar surface appearance.

### 3.2. Loss on Drying of B Cellulose

The moisture content of the borojó cellulose isolate was 4.38%.

### 3.3. Rheological Properties of Excipients

#### 3.3.1. Carr’s Index and Hausner’s Ratio

After getting the values for the poured and tapped density three times, we calculated the Carr’s index and Hausner’s ratio using Equations (4) and (5). The averages for these properties are shown in Table 2.

An ANOVA test for the Carr’s index gave a *p*-value of 0.0003, which is smaller than the significance level of 0.01. Thus, the results demonstrate that the Carr’s index for the different cellulose types is not equal. As said in the methodology section, to know which ones were equal, we used Tukey multiple comparisons of means with a 95% family-wise confidence level. The *p*-values were as follows: 0.0018402 for CC-BC, 0.1516694 for CMCC-BC, and 0.0003633 for CMCC-CC. The only one that shows a *p*-value greater than the significance level is CMCC-BC, meaning that they are not statistically significantly different.

A statistical test for Hausner’s ratio showed a *p*-value of 0.0003, which is smaller than the significance level 0.01. This means that the different cellulose types are not all equal. The results of the Tukey multiple comparisons of means with a 95% family-wise confidence level were as follows: 0.0025 or CC-BC, 0.0916 for CMCC-BC, and 0.0003 for CMCC-CC. CMCC-BC is the only one that shows a *p*-value greater than the significance level, meaning that they are not statistically significantly different.

#### 3.3.2. Angle of Repose

The values of the angle of repose calculated three times using Equation (6) are represented in Table 3. The values of CMCC were set for the maximum value (41°) as there was no flow.

An ANOVA test performed using *R* Studio gave a *p*-value of 1.41e^−06^ ***, smaller than the significance level 0.01, thus we can say that the angle of repose for the different cellulose types are not all equal.

Tukey multiple comparisons of means gave *p*-values of 0.0005 for CC-BC, 0.0000013 for CMCC-BC, and 0.000014 for CMCC-CC. All *p*-values are smaller than the significance level, meaning that they are all are statistically significantly different.

### 3.4. Fabrication and Quality Control of Tablets

#### 3.4.1. Hardness, Friability and Disintegration

**Figure 4:**
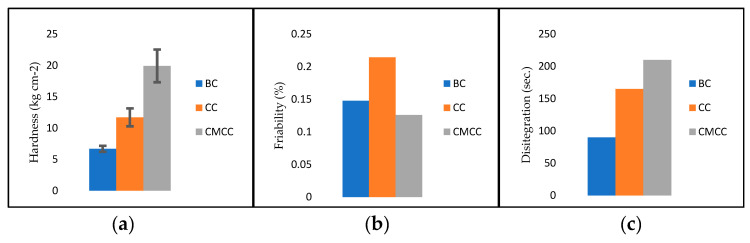
Results of quality control tests for the tablets. (**a**) Hardness for tablets with BC, CC, and CMCC with standard deviation. (**b**) Friability or percentage of weight loss of acetaminophen tablets with each excipient. (**c**) Disintegration time of tablets at 37 °C with water as medium.

A hardness test was executed to assure that the tablet will not break easily through the processes of manufacturing, packaging, and transportation. It is also essential to make sure that it is not too hard as it can affect the disintegration and dissolution of the drug. Results obtained directly from the durometer, repeated six times, were averaged (Figure 4a). It can be seen that borojó cellulose shows the lowest values, followed by commercial cellulose and then commercial microcrystalline cellulose.

For friability, the results of the individual weight of six tablets containing BC, CC, and CMCC compared with the weight after placing them in the friabilator were used to obtain the values by applying Equation (7). The most resistant tablets are composed of CMCC, but followed closely by BC. Figure 4b shows that none of the values are higher than 1%, meaning that all the tablets are acceptable for solid dosage of a pharmaceutical.

The disintegration time experiment aimed to determine the time at which the tablets break up into small granules by swelling of water in the cellulose fiber structure. This is important to mimic the process by which the tablet enters the body and disintegrates to release the drug [25]. The values of the time at which the tablets disintegrated for each case are represented in Figure 4c. We observed that borojó cellulose is the one that disintegrates first, followed by commercial cellulose, and then commercial microcrystalline cellulose.

#### 3.4.2. Acetaminophen Dissolution Test

**Figure 5:**
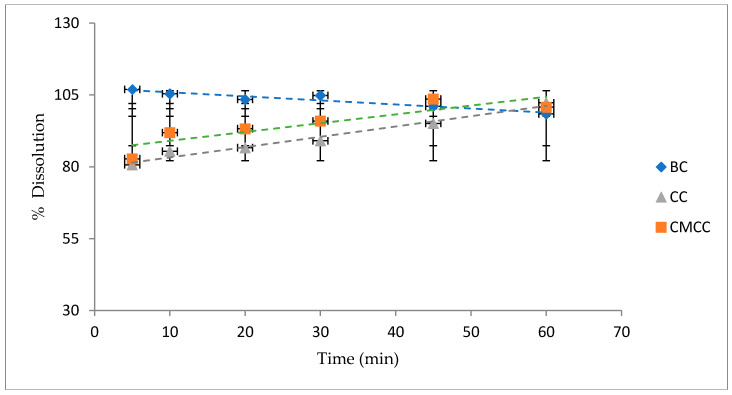
Dissolution profile of acetaminophen from tablets with BC, CC, and CMCC as excipients at 37 °C with 0.05 M monobasic phosphate buffer pH 5.8 as medium.

From an initial percentage loading of acetaminophen in each tablet of 10% w/w, we determined the percentages of the model drug dissolved in each fraction after 5, 10, 20, 30, 45, and 60 min (Figure 5). The dissolution profile of acetaminophen up to 60 min from the tablets with BC (blue diamonds), CC (gray triangles), and CMCC (orange squares) are plotted. The results suggest that BC tablets dissolve faster than CC and CMCC tablets. Nevertheless, all the tablets release 80% or more of the acetaminophen in the first 5 min, the typical behavior of an immediate release tablet.

## 4. Discussion

In the present study we aimed to evaluate microcrystalline cellulose extracted from the native Ecuadorian plant, borojó, as an excipient for solid dosage tablets and compare it with commercial cellulose. Results from Figure 1 suggest that the powders with higher crystallinity are microcrystalline cellulose and borojó cellulose, as they have bigger and sharper peaks compared to the diffuse peaks of commercial cellulose [26]. It is possible that contamination could come from the metallic spatula used to collect and transfer the cellulose since we might have used a different spatula for all samples. It is also possible that it is due to the “crystallization” when in contact to some salts like NaCl, etc. However, the results show that BC has the lowest crystallinity index of 44.1% compared to both commercial celluloses. This may be due to the different source of cellulose that can affect the crystallinity index or the extraction process [27]. It is expected that the crystallinity of the excipient could affect the properties of the tablet.

Considering the structure of the compounds, according to the FTIR spectra in Figure 2, all the graphs show similar characteristic absorption peaks, which means that the same functional groups are present in the extracted cellulose and commercial cellulose compounds [26]. Therefore, all the samples are mostly composed of cellulose, which is critical for comparing the excipients’ properties. 

From the results of SEM in Figure 3, a difference in size of the particles of powders of BC, CC, and CMCC are apparent. CMCC shows the smallest average particle followed by BC, and then CC. Previously, particle size has been used as a parameter to judge flow rate [28]. One would expect that flow is faster when the particle size is larger, although other factors like crystallinity can also have an effect. This property is important because it influences many factors of drug manufacturing such as mixing and tableting machine performance. A faster flow will allow powders to combine better, and the compression machine will produce a higher number of tablets per time unit [29,30].

We confirmed that the value obtained for loss on drying for BC was low, just 4.38%. Moreover, this result is within the values accepted by the European Pharmacopeia, which states that moisture content should not exceed 7.0% (m/m) to be used as an excipient [31]. On the other hand, rheologic results compared with existent values were within acceptable ranges, at least between fair to good [18]. This represents potential to be used for compression fabrication as fluidity is a very important characteristic. Moreover, from the statistical analysis, we can say that the results of BC are more similar to CMCC.

After compression, the quality control tests were applied. Hardness, shown in Figure 4a, gave higher results for CMCC, followed by CC, and then BC. All results were above 5 kg cm^−2^. Normally, this value could represent sufficient hardness to prevent breakage during handling and transportation but should disintegrate and dissolve without problems [32]. The different hardness values between the excipients are expected to be due to the different crystallinity indexes. The other indicator of tablet strength that was evaluated was friability (Figure 4b), with values below the accepted limit, 1%. [32]. Dyatlov has reported the friability of four different types of paracetamol tablet compared to a reference tablet. All tablets had a friability value less than 1%, which is consistent with our results [33]. The last physical test was disintegration (Figure 4c). The maximum time that can be seen in the graph is well below the established value of less than 15 min for uncoated tablets [21].

The dissolution profile showed a fast release of the drug from the tablets formulated with BC as compared to the other excipients. Moreover, it is within the specifications established by USP for acetaminophen dissolution, which state that it must be greater than or equal to 80% in 30 min [23]. A range of 95–105% of content is usual, and 90–110% is permitted in US Pharmacopeia. The amount released could decrease with time due to several factors: degradation, adsorption, absorption, precipitation, etc. The rapid release is similar to the reference paracetamol tablet data reported by Dyatlov [33]. The difference between BC and both commercial cellulose at early time points is within the range of 20%, which could be critical to demonstrate bioequivalence. There is no clear trend of the effect of the particle size on the release. The lower crystallinity of BC compared with its commercial options may explain this difference in dissolution. Amorphous solids typically possess a bigger energetic content, so they are solvated faster [34].

## 5. Conclusions

According to the characterization techniques, borojó cellulose shows a similar structure, shape, and size as compared to commercial microcrystalline cellulose. Although the BC crystallinity index was the lowest of the three samples, it seems to have a good crystalline content. Powders of the extracted cellulose also showed good characteristics and acceptable values of moisture content and fluidity, which positions them with good potential to be used as an excipient. The quality tests on the final tablets showed that tablets composed of borojó cellulose excipient could be used without any problem of fracture, abrasion, or lack of disintegration.

Finally, the dissolution profile showed that the tablets with the three excipients release the active substance according to the values established by the United States Pharmacopoeia, but tablets with borojó cellulose deliver the drug faster. Thus, its use may be appropriate for immediate-release tablets.

For future investigations it should be considered to test borojó cellulose for use as an excipient for immediate-release tablets or to use cellulose extracted from other native plants with a higher crystallinity index to explore the diversity these would give as excipients in pharmaceutical applications.

## Figures and Tables

**Table 1:** Constituents of blend for compression.

Component	Function	Amount Used (%)	Mass Used (g)
Acetaminophen	Active Principle	10	2
Cellulose	Diluent/disintegrant	35	7
Lactose	Diluent/binder	53.75	10.75
Aerosil	Glidant	0.25	0.05
PEG 600	Lubricant	1	0.2

**Table 2:** Carr’s index and Hausner’s ratio average for all three excipients.

	B Cellulose	Commercial Cellulose	Commercial MCC
Carr’s index	16.65	12.37	18.15
Hausner’s ratio	1.19	1.14	1.22

**Table 3:** Angle of repose for BC, CC, and CMCC.

BC	CC	CMCC
25.38	31.78	41
25.27	29.05	41
24.26	30.6	41

## References

[1] Pharmaceutical Outsourcing Solid Dosage Processing and Packaging. https://www.pharmoutsourcing.com/Featured-Articles/350152-Solid-Dosage-Processing-and-Packaging/.

[2] Hamman J.H., Tarirai C. (2006). Functional excipients. Chem. Today.

[3] IPEC (2008). The Joint IPEC-PQG Good Manufacturing Practices Audit Guideline for Pharmaceutical Excipients.

[4] Zatz J., Kushla G., Banker G.S., Rieger M.M., Lieberman H.A. (1996). Pharmaceutical Dosage Forms: Disperse Systems.

[5] Pal R.S., Pal Y., Wal A., Wal P. (2019). Current Review on Plant based Pharmaceutical Excipients. Open Med. J..

[6] Balandrin M., Klocke J., Wurtele E., Bollinger W. (1985). Natural plant chemicals: Sources of industrial and medicinal materials. Science.

[7] Singh P., Mahmood T., Shameem A., Bagga P., Ahmad N. (2016). A review on Herbal Excipients and their pharmaceutical applications. Sch. Acad. J. Pharm..

[8] Shokri J., Adibki K., Van De Ven T.G.M. (2013). Application of Cellulose and Cellulose Derivatives in Pharmaceutical Industries. Cellulose—Medical, Pharmaceutical and Electronic Applications.

[9] Lee C.K., Diesendruck C.E., Lu E., Pickett A.N., May P.A., Moore J.S., Braun P.V. (2014). Solvent swelling activation of a mechanophore in a polymer network. Macromolecules.

[10] Sun B., Zhang M., Shen J., He Z., Fatehi P., Ni Y. (2019). Applications of Cellulose-based Materials in Sustained Drug Delivery Systems. Curr. Med. Chem..

[11] Nobles D.R., Romanovicz D.K., Brown R.M. (2001). Cellulose in cyanobacteria. Origin of vascular plant cellulose synthase?. Plant Physiol..

[12] Brown R.M., Wiessner W., Robinson D.G., Starr R.C. (1990). Algae as tools in studying the biosynthesis of cellulose, nature’s most abundant macromolecule. Cell Walls and Surfaces, Reproduction, Photosynthesis.

[13] Kimura S., Itoh T. (1998). A new cellulosic structure, the tunic cord in the ascidian Polyandrocarpa misakiensis. Protoplasma.

[14] Trache D., Hussin M.H., Hui Chuin C.T., Sabar S., Fazita M.R.N., Taiwo O.F.A., Hassan T.M., Haafiz M.K.M. (2016). Microcrystalline cellulose: Isolation, characterization and bio-composites application—A review. Int. J. Biol. Macromol..

[15] Kulkarni G.T., Gowthamarajan K., Dhobe R.R., Yohanan F., Suresh B. (2005). Development of controlled release spheriods using natural polysaccharide as release modifier. Drug Deliv..

[16] Morán J.I., Alvarez V.A., Cyras V.P., Vázquez A. (2008). Extraction of cellulose and preparation of nanocellulose from sisal fibers. Cellulose.

[17] Robles L.V. (2011). Los excipientes y su funcionalidad en productos farmacéuticos sólidos. Revista Mexicana de Ciencias Farmacéuticas.

[18] Días C., Camacho A. (2005). Elaboración de Productos Farmaceúticos y Parafarmaceúticos.

[19] Montanari D., Agostini A., Bonini M., Corti G., Del Ventisette C. (2017). The use of empirical methods for testing granular materials in analogue modelling. Materials.

[20] Pharmapproach Quality Control Tests for Tablets. https://www.pharmapproach.com/quality-control-tests-for-tablets/2.

[21] Al-Gousous J., Langguth P. (2015). Oral Solid Dosage Form Disintegration Testing—The Forgotten Test. J. Pharm. Sci..

[22] The United States Pharmacopeia (USP) (2016). Monograph: Dissolution.

[23] Pèrez López E. (2014). “In vitro” solution test of acetaminophen tablets, hplc quantifying with electrochemical detector. Intersedes.

[24] Klemm D., Heublein B., Fink H.P., Bohn A. (2005). Cellulose: Fascinating biopolymer and sustainable raw material. Angew. Chem. Int. Ed..

[25] Markl D., Zeitler J.A. (2017). A Review of Disintegration Mechanisms and Measurement Techniques. Pharm. Res..

[26] Chandran I.S., Prasanna P.M. (2015). Drug-excipient interaction studies of loperamide loaded in polsorbate 80 liposomes. Orient. J. Chem..

[27] Bravo I., Figueroa F., Swasy M.I., Attia M.F., Ateia M., Encalada D., Vizuete K., Galeas S., Guerrero V.H., Debut A. (2020). Cellulose Particles Capture Pollutants. RSC Adv..

[28] Clerch A. (2008). Aportación al Diseño de un Nuevo Excipiente Tipo “Coprocessed Product” Para Compresión Directa. Doctoral Thesis.

[29] Cornejo L., Cordero M. (2007). Evaluación de las Propiedades Farmacotécnicas en el Diseño y Formulación de Tabletas de Clorfenamina por Compresión Directa. Bachelor’s Thesis.

[30] Bordallo J.S. (1994). Determinación por Hplc de 4-Epianhidro-Tetraciclina Como Producto de Degradación en Formas Orales de Tetraciclina Condicionada por Nuevos Diseños Galenicos. Bachelor’s Thesis.

[31] Council of Europe (2010). European Pharmacopoeia European Pharmacopoeia 7.0.

[32] Gad S.C. (2008). Pharmaceutical Manufacturing Handbook: Production and Processes.

[33] Dyatlov N.A. (2018). Pharmaceutical formulation and investigations of sustained-release paracetamol matrix tablets and their potential antipyretic effect in children. Sci. Pract. J. Obstet. Gynecol..

[34] Vila J.L. (2001). Tecnología Farmacéutica: Formas Farmaceúticas.

